# Surgery

**Published:** 1895-09

**Authors:** James Swain


### SURGERY.

The subjects chosen for discussion in the Surgical section at the recent meeting of the British Medical Association proved instructive, more from the fact that they emphasised trustworthy methods of treatment which are too little known, rather than from the advancement of any very startling theories or therapeutic measures. The progress of knowledge is always slow, and it is a question whether the enormous mass of literature which now floods our libraries has not defeated its object by preventing the busy practitioner from separating the good, bad, and indifferent. Thus it is that we are content to go on in "the old sweet way," too often unconscious that the old order has been changed.

Sir William Stokes introduced the subject of the treatment of fractures of the upper third of the femur, and the chief point of interest was in his remarks on the treatment of impacted fractures of the neck of the thigh bone associated with considerable deformity and shortening. There has generally been a wide-spread feeling against interference with impacted fractures because it was supposed that in these cases the fragments were in a better condition for union than when dis-impacted so as to permit a separation of the fragments. In other situations most of us have long ago ignored this crude principle—notably in the case of Colles's fracture, where the deformity is rendered less and the utility greater when the impaction of the fragments is forcibly overcome—and Sir William Stokes, Mr. Bryant, and others, have shown that under certain circumstances there is no reason why in cases of impacted fracture of the neck of the femur the fragments should not be forcibly separated and the limb brought to its proper length and position. In the case of the aged, it may not be wise to subject the tissues to a further damage than that caused by the injury, but in a young and vigorous adult we should not be content to leave the impacted fragments to become united in a distorted position, with consequent shortening and lessened usefulness of the limb, when, by purposely overcoming the impaction, the injured limb may recover with complete restoration of its former length and utility —and this is what should be aimed at. Union of fragments depends mainly on their correct apposition, and this can be secured with due care in both impacted and non-impacted fractures. This statement, of course, applies with equal truth to the so-called "intra-capsular fractures" of old age which really unite —if we treat them as if we expected to get union—in a more satisfactory manner than one would suppose by reading the various text-books. Indeed, speaking generally, there is no need to treat these "intra-capsular" fractures in a different manner to the so-called "extra-capsular" fractures, and it would be better to agree with Bryant and avoid this division of fractures of the neck of the femur into the intra- and extra-capsular varieties.

In the second subject chosen for general discussion, Mr. Butlin in introducing the debate on the Surgical Treatment of Tumours of the Thyroid Gland, drew attention to the fact that there is too great a tendency to regard these growths as affecting the whole, instead of a portion only, of the gland. It would be just as absurd to speak of an "adenoma of the breast" as an "enlargement of the breast," as it is to adopt the usual term of "hypertrophied thyroid" where we should really speak of an "adenoma of the thyroid." It is of the greatest importance to recognise the fact that in a large number of thyroid enlargements only a portion of the gland is affected by a distinctly encapsuled growth, and when this has been "shelled out" from its capsule—which is the best method of treatment in these cases—the rest of the gland need not be interfered with. The removal of large and indefinite portions of the thyroid gland in cases of benign tumour is unnecessary, and is not analogous to our treatment of similar tumours elsewhere. It is on this point that Mr. Butlin rightly insisted. Certainly, as Mr. Mayo Robson pointed out, the encapsulation is not always so distinct as Mr. Butlin's paper would make it appear, but it nevertheless behoves us to treat these tumours on a rational basis, although exceptional cases may require us to depart from our orthodox rules. Most English surgeons would prefer to perform the operation under an anæsthetic, and chloroform is generally chosen as tending to cause less congestion of the vessels of the neck than ether ; but Professor Keen reminds us that Professor Kocher, and other Continental surgeons, are in the habit of removing large thyroid tumours without any anæsthetic, and apparently without causing the patient much pain. A vertical incision, supplemented, if needs be, by a transverse one, is usually adopted in England, but on the Continent a transverse incision (afterwards drawn together by subcutaneous suture) is favoured, because the subsequent scarring is less seen.

Professor Murphy, by a demonstration on the cadaver of the use of his button, once more brought to mind the unsettled question of Intestinal Anastomosis. The fact that so many different kinds of sutures and mechanical means have been devised for the purpose of approximating divided intestine shows more than anything else that we have not yet got an ideal method, and although Murphy's button has rendered the operation simple and effective, there are many grave objections to its use, and it in turn will no doubt be superseded by other methods. The statistics[1] of recorded cases in which the Murphy button has been used are remarkable for the great success attending its employment, and Murphy[2] himself asserts his belief in the superiority of his device over all others. Treves[3], too, speaks highly of the button. On the other hand, Senn believes in his absorbable plates, Mayo Robson in his "bone-bobbin," Von Baracz and Dawbarn in vegetable plates, and König and others discard all forms of apparatus and rely only on suturing. Dawbarn,[4] in discussing the relative value of the Murphy button and absorbable plates, maintains that, with the exception of the operation of cholecystenterostomy, approximation by means of the Murphy button gives inferior results to those obtained by such absorbable plates, and supports his argument by quoting several unsuccessful cases where the Murphy button was used, and by referring to an article by Magill,[5] in which the statistics are discussed in detail. Dawbarn has always insisted on the superiority of raw vegetable (turnip) plates over those made of decalcified bone, though of course the principle remains the same in each; and certainly the

[1] *Med. News*, vol. lxvi., 1895, p. 141, and *Practitioner*, [vol. liv.,] 1895, p. 454.
[2] *Lancet*, 1895, vol. i., p. 1040. [3] *Practitioner*, [vol. liv.,] 1895, p. 481.
[4] *Ann. Surg.*, vol. xxi., 1895, p. 166. [5] *Ibid.*, vol. xx., 1894, p. 297.

vegetable plates have the advantage of being cheap, always obtainable, and of more rapid absorption, so that they are less likely to cause obstruction. The most rigid test on this point is, of course, a gastro-enterostomy; and Von Baracz's frequent successes with such plates, in this operation, speak for themselves. König[1] thinks that the use of the Murphy button, by enabling surgeons of less experience to operate, is by no means an advantage to the patient; and although he recognises the fact that the time occupied in the operation of intestinal anastomosis may be shortened by the use of the various mechanical means we have referred to, believes that a prolonged laparotomy does not lead to shock. He therefore still uses the method of suture which he considers has been shown to be safe, instead of the more modern methods which, though rapid, seem to him of doubtful safety. Amidst this conflicting testimony a large discount must be allowed for individual bias. Most surgeons will be disinclined to agree with König as to the influence of a prolonged abdominal operation in producing shock, and, therefore, we are constrained to admit that there are instances in which we believe that mechanical methods have been the means of saving life, when used in cases that have been too exhausted to permit of the extra time demanded by accurate suturing. Those who have seen suturing plates, bobbins, and buttons used, have been struck with the simplicity and rapidity with which the invention of Dr. Murphy can be applied, and in this its superiority seems to rest. As I have already said, there are grave objections to its use in intestinal anastomosis—of which only I am speaking. The chief of these, if the button has been properly applied, consist in the possible blocking of the button itself while it remains *in situ*, and the secondary lodgment of the button in the ileo-cæcal valve, causing symptoms of obstruction. The latter of these is the more important, but can largely be avoided by a selection of a button of the proper size. In the large intestine this accident, of course, cannot happen, and here, at all events, we may, in ordinary cases, use the button with a good prospect of success. It must not be thought that the button can be used indiscriminately, even in the large intestine, and especially in cases of obstruction due to malignant disease, the merits of the case should be carefully weighed before resorting to its use. Indeed, in these cases, it is probably best, as advocated by Mr. Greig Smith, to operate in two stages. Intestinal surgery does not admit of the exclusive adoption of any one method, and the best results will be obtained by varying the means at our command, according to the needs of the individual case. The surgeon who feels competent to perform intestinal anastomosis by means of Murphy's button only had better not undertake the operation. This clever device is worthy of much praise, but it does not admit of universal

[1] *Brit. M. J.*, 1895, vol. i., Epitome, p. 26.

application, and can only be regarded as *one* of the best means we at present possess for the union of divided intestine.

The simplicity and celerity in the application of the Murphy button depend largely on the fact that in placing each half of this instrument in the divided ends of the gut we have only to take what is known as a "purse string" stitch around each end of the intestine. This can be done very quickly, and leaves us with only one thread to tie. The final approximation of the upper and lower ends of the gut is then accomplished by pressing the two halves of the button together. In the various bone bobbins and tubes which have been devised a more elaborate system of stitching is generally necessary, and hence the operation is not usually so quickly performed as with the Murphy button; but the latest addition to the "bobbins" is as easily placed in the gut as Murphy's button, and this, though it is too new to have established any reputation, we feel bound to notice before finally quitting the subject. The decalcified bone bobbin of Mr. Herbert Allingham[1] has the form of two truncated cones joined end to end. The narrowest portion of the bobbin is therefore in the middle, and it follows that if purse string stitches be drawn tightly round the two ends of the divided gut they must each tend to slide to the middle of the bobbin, and therefore become approximated; and if the opposing portions of the intestinal peritoneum be scarified, plastic union will occur. In order to facilitate the firm tying of the purse string stitches the central part of the bone bobbin is not decalcified. This bobbin seems a step in the right direction, but in the absence of any practical experience in its use further criticism must be postponed.

Another of the unsettled questions in abdominal surgery is the treatment of intra-abdominal lesions, the fatality of which is notorious. Here again we have the usual divergence of opinion, largely owing to the fact that a correct diagnosis is extremely difficult, for in these cases we find comparatively slight external evidences of injury, although the general condition of the patient is suggestive of a serious internal lesion. Deaver[2] thinks that the majority of these cases demand abdominal section; although, except in extreme cases, he would not advise it unless the diagnosis was tolerably certain. Chaput,[3] Berger,[4] and Michaux[5] are also in favour of early intervention. Michaux regards it as certain that an exploratory laparotomy should be at once undertaken in severe contusions of the abdomen. He insists that good results can only be ensured by early and rapid operation. Of six patients on whom he has

[1] *Lancet*, 1895, vol. ii., p. 518.
[2] *Ann. Surg.*, vol. xxi., 1895, p. 326. [3] *Med. Week*, vol. iii., 1895, p. 57.
[4] *Ibid.*, p. 68. [5] *Ibid.*, pp. 152, 177.

operated within the first twenty-four hours none died; but of four cases, in which operation was delayed to the twenty-third and twenty-sixth hour, third and seventeenth day respectively, two died. Sorge[1] writes in a similar strain, and refers to fourteen cases of abdominal injury (stabs, gun-shot wounds, contusions) in which he performed laparotomy as soon as possible, and of whom nine recovered. Battle[2] is of opinion that operation should be resorted to if the shock is considerable or increasing, and associated with a large or increasing amount of blood in the peritoneal cavity. In favour of a more expectant plan of treatment we find Berger,[3] Manley,[4] and Chaput.[5] The last-mentioned thinks that we should always abstain from intervention in cases of severe shock, unless there are such distinct symptoms of hemorrhage as to demand operation; but he considers that an operation is indicated by a previously existing shock, accompanied by abdominal pain, retraction of the abdomen, nausea, and bilious vomiting. Delorme[6] refers to nine cases of severe contusion of the antero-lateral walls, of which two only were subjected to operation: the other seven spontaneously recovered. Mr. Bryant[7] recorded twenty-seven cases of severe abdominal contusion treated on the expectant plan (for it was before abdominal operations were thought of in these cases), and all recovered except one. In summing up these various opinions, it seems pretty clear that except in very severe cases exploratory operations are unjustifiable. It is well-known that blows on the epigastrium are capable of producing the most severe symptoms, without causing any distinct lesion; and the knowledge of this fact alone should make us cautious in too hastily deciding to operate in the presence of mere shock. Where there are symptoms of severe hemorrhage or laceration of a viscus, our course is clearly to operate without delay; but in more dubious cases we can afford to wait and see whether the symptoms are improving or getting worse—a point which can generally be decided under twenty-four hours—and then plan our treatment accordingly. Manley[8] truly observes of these cases: "Laparotomy, serious as it is and always must be . . . . does occasionally offer the only possible hope of saving life, and for such should be recommended."

JAMES SWAIN.

### DERMATOLOGY.

In his Presidential address[9] before the Dermatological Society of Great Britain and Ireland, Dr. Pye-Smith said that unless pursued by scientific methods and on pathological principles, dermatology would again, he thought, become, what it once

1 *Brit. M. J.*, 1895, vol. i., Epitome, p. 82.

2 *Lancet*, 1895, vol. i., p. 219. 3 *Med. Week*, vol. iii., 1895, p. 68.

4 *Therap. Gaz.*, vol. xix., 1895, p. 12. 5 *Med. Week*, vol. iii., 1895, p. 161.

6 *Ibid.* 7 *Guy's Hosp. Rep.*, 1861, p. 30. 8 *Loc. cit.*

